# Added value and cascade effects of inflammatory marker tests in UK primary care: a cohort study from the Clinical Practice Research Datalink

**DOI:** 10.3399/bjgp19X704321

**Published:** 2019-06-18

**Authors:** Jessica Watson, Chris Salisbury, Penny Whiting, Jonathan Banks, Yvette Pyne, Willie Hamilton

**Affiliations:** Centre for Academic Primary Care, Bristol Medical School, and the National Institute for Health Research (NIHR) Collaboration for Leadership in Applied Health Research and Care West (CLAHRC West), University of Bristol, Bristol.; Centre for Academic Primary Care, Bristol Medical School, and the National Institute for Health Research (NIHR) Collaboration for Leadership in Applied Health Research and Care West (CLAHRC West), University of Bristol, Bristol.; Centre for Academic Primary Care, Bristol Medical School, and the National Institute for Health Research (NIHR) Collaboration for Leadership in Applied Health Research and Care West (CLAHRC West), University of Bristol, Bristol.; Centre for Academic Primary Care, Bristol Medical School, and the National Institute for Health Research (NIHR) Collaboration for Leadership in Applied Health Research and Care West (CLAHRC West), University of Bristol, Bristol.; Centre for Academic Primary Care, Bristol Medical School, University of Bristol, Bristol.; University of Exeter Medical School, University of Exeter, Exeter.

**Keywords:** diagnosis, inflammatory markers, primary care

## Abstract

**Background:**

Inflammatory markers (C-reactive protein, erythrocyte sedimentation rate, and plasma viscosity) are commonly used in primary care. Though established for specific diagnostic purposes, there is uncertainty around their utility as a non-specific marker to rule out underlying disease in primary care.

**Aim:**

To identify the value of inflammatory marker testing in primary care as a rule-out test, and measure the cascade effects of testing in terms of further blood tests, GP appointments, and referrals.

**Design and setting:**

Cohort study of 160 000 patients with inflammatory marker testing in 2014, and 40 000 untested age, sex, and practice-matched controls, using UK primary care data from the Clinical Practice Research Datalink.

**Method:**

The primary outcome was incidence of relevant disease, including infections, autoimmune conditions, and cancers, among those with raised versus normal inflammatory markers and untested controls. Process outcomes included rates of GP consultations, blood tests, and referrals in the 6 months after testing.

**Results:**

The overall incidence of disease following a raised inflammatory marker was 15%: 6.3% infections, 5.6% autoimmune conditions, 3.7% cancers. Inflammatory markers had an overall sensitivity of <50% for the primary outcome, any relevant disease (defined as any infections, autoimmune conditions, or cancers). For 1000 inflammatory marker tests performed, the authors would anticipate 236 false-positives, resulting in an additional 710 GP appointments, 229 phlebotomy appointments, and 24 referrals in the following 6 months.

**Conclusion:**

Inflammatory markers have poor sensitivity and should not be used as a rule-out test. False-positive results are common and lead to increased rates of follow-on GP consultations, tests, and referrals.

## INTRODUCTION

Inflammatory marker tests include C-reactive protein (CRP), plasma viscosity (PV), and erythrocyte sedimentation rate (ESR). Millions of inflammatory marker tests are done annually, and rates of testing are rising,^1^ with large variation in testing rates between different GP practices.^2^ Measurement of inflammatory markers has two functions; it contributes to diagnosis of inflammatory conditions, including infections, autoimmune conditions, and cancers, and it is used to monitor disease progression or treatment response.^3^ Inflammatory markers are recommended in a limited number of national guidelines, for example, as a first-line test for myeloma,^4^ polymyalgia,^5^ and pneumonia.^6^

A third use has crept into clinical practice; as a non-specific test to rule out serious underlying disease and provide patient and GP reassurance.^7^ Patients with non-specific symptoms, such as tiredness, memory problems, or gastrointestinal symptoms, may have inflammatory marker testing performed in order to exclude other diagnoses, as recommended in guidelines for chronic fatigue, dementia, and irritable bowel syndrome.^8^^–^10 There is a lack of evidence to back up this clinical practice. Unexpected results can be challenging to interpret, and false-positives may lead to increased uncertainty and anxiety for patients and GPs, and a cascade of further tests.^11^ False-negatives may lead to false reassurance and delayed diagnosis of underlying diseases.

The concept that abnormal test results can lead to cascade testing is not new,^12^^,^^13^ yet little evidence of the frequency of cascade testing exists.^14^ Potential overuse of pathology tests is important given the current financial constraints within the NHS.

Much of the evidence about inflammatory markers comes from secondary care, and focuses on single disease outcomes.^3^ This is not helpful when testing is done for non-specific symptoms, where multiple diseases are possible. The aim of this study was to identify the value of inflammatory marker testing in primary care as a rule-out test, provide evidence for GPs to interpret inflammatory markers, and to measure the cascade effects of testing in terms of follow-on blood tests, GP appointments, and referrals.

## METHOD

### Participants and data sources

This was a prospective cohort study using the Clinical Practice Research Datalink (CPRD), which contains anonymised routinely collected data recorded from primary care electronic health records. Participants were 160 000 patients aged >18 years with an inflammatory marker taken in 2014. The inflammatory markers considered were CRP, ESR, and PV. The index date was defined as the first date of inflammatory marker testing in 2014.

**How this fits in**

The utility of inflammatory markers as a general but non-specific test for possible serious underlying disease in primary care is poorly understood. In this large observational study using UK primary care electronic health records, the authors found that the most common cause of raised inflammatory marker is infection (6.3%), followed by autoimmune conditions (5.6%), and cancers (3.7%). Inflammatory markers have poor sensitivity, and should therefore not be used as a rule-out test. False-positive inflammatory marker results are common, and are associated with increased rates of follow-on GP consultations, tests, and referrals.

**Figure 1.**
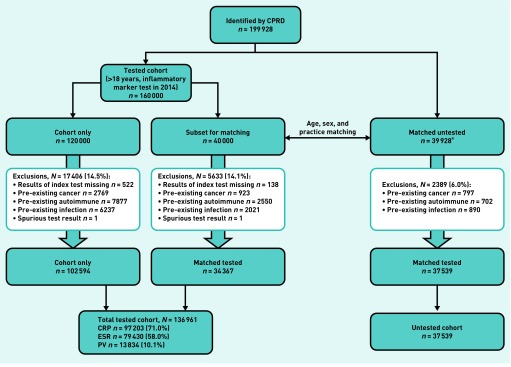
***Flowchart showing exclusions. CPRD = clinical practice research datalink. CRP = C-reactive protein. ESR = erythrocyte sedimentation rate. PV = plasma viscosity.^a^Matched untested consists of 39 928 subjects because, of the 40 000 from the cohort who were randomly selected for matching, 72 had no suitable age, sex, and practice-matched control.***

A comparison sample of 40 000 patients had no inflammatory marker test in 2014, though could have had testing at other dates. These were matched by age (in 5-year bands), sex, and practice to a random subset of 40 000 patients from the inflammatory marker test group. Controls were allocated the same index date as their matched case. Patients who had received a diagnosis of cancer, autoimmune conditions, or chronic infections in the 2 years before the index date were excluded, as were patients with an acute infection in the 30 days before the index date (Figure 1).

Linked data included English Cancer Registry Data and patient level index of multiple deprivation (IMD). The authors did not solely study participants with data linkage in case this introduced bias. Cancer registry data were available for 110 245 patients, IMD for 110 181.

### Index tests

The authors defined a raised inflammatory marker using the mean upper limit of normal from laboratories within the study. For CRP, it was 6.8 mg/l, for simplicity rounded to 7 mg/l; for PV it was 1.72 mPa/s. For ESR, it was stratified by sex and age (further information is available from the authors on request). When the same inflammatory marker was repeated on the same day (*n* = 231), the authors retained the highest value. A binary variable for any raised inflammatory marker was generated to accommodate multiple testing.

### Outcome variables

The primary outcome was any relevant disease, defined as cancer or autoimmune conditions coded within 1 year, or infection within 1 month of the index date. The authors considered infections beyond 1 month unlikely to relate to the initial raised inflammatory marker. Process outcomes of repeat GP consultations, additional blood tests, and referrals were identified. For consultations, the authors included face-to-face, home visit, and telephone consultations.

### Code list development

To identify cancers, the authors used validated code lists used in multiple previous studies (further details are available from the authors on request), as well as Cancer Registry Data. Clinical code lists for infections and autoimmune conditions were developed using validated methods,^15^ are broken down into subtypes (further information is available from the authors on request), and are available on the University of Bristol Data Repository.^16^

The authors searched the CPRD for symptom codes in the 28 days before and including the index date, retaining the 200 most frequently occurring codes, categorised according to the International Classification of Primary Care.^17^ They then used methods described previously^15^ to generate complete code lists for each of these symptoms.

### Sample size calculation

The CPRD were willing to offer a sample size of 160 000 tested patients. Power calculations with α = 0.05 and assuming that one-third of tests would be abnormal, with a baseline incidence of relevant disease of 7.5% (1% cancers, 1.5% autoimmune, 5% infections), gave 93% power to identify a change in incidence to 8%. For the least frequently occurring disease category, cancer, the authors had a 94% power to identify an increase from 1% to 1.2%. As the focus of the study was disease outcomes in tested patients, the untested group, used as a benchmark for the tested group, was deliberately kept small to ensure maximum power in the main study.

### Analysis

The primary analysis measured the overall incidence of relevant disease for patients with raised versus normal inflammatory markers, as well as tested versus untested patients, equivalent to the positive predictive value (PPV) in the test-positives. The authors used logistic regression for the dose–response relationship between CRP, ESR, and PV as continuous variables, and relevant disease as a binary variable, also generating a receiver-operating characteristic (ROC) curve. Summary statistics including sensitivity, specificity, PPV, and negative predictive value (NPV) were calculated for each of the three inflammatory markers. For ease of reading, the authors present many of the results for a primary care population of 1000. The reporting of this study conforms to the STARD^18^ and RECORD statements.^19^ Analysis was performed using Stata (version 15).

## RESULTS

### Tests requested

After exclusions (Figure 1), the sample included 136 961 patients with one or more inflammatory marker test: 71.0% CRP, 58.0% ESR, and 10.1% PV, plus 37 539 untested. More than one inflammatory marker was performed simultaneously on the index date in 38.8%. Of the overall tested cohort, 27.8% had one or more raised inflammatory marker.

### Patient demographics

Compared to the UK adult population, the tested cohort were more likely to be female (61.6% versus 51.3%), of white ethnicity (87.0% versus 85.4%), and from the most affluent socioeconomic quintile (23.0% versus 20%) (further information is available from the authors on request). Raised inflammatory markers were more common among the most deprived socioeconomic quintile (odds ratio [OR] 1.31, 95% confidence interval [CI] = 1.24 to 1.38, *P*<0.001), and more common among females (OR 1.19, 95% CI = 1.16 to 1.22, *P*<0.001). There was no difference in the frequency of abnormal results by ethnicity.

### Incidence of disease

The overall incidence of disease in patients with a raised inflammatory marker (PPV) was 15.0% — 6.3% infections, 5.6% autoimmune conditions, and 3.7% cancers (Table 1).

**Table 1.** Disease incidence according to inflammatory marker test results

		**Any relevant disease, *n* (%)**	**Autoimmune disease, *n* (%)**	**Infections, *n* (%)**	**Cancer, *n* (%)**
**Untested (*n*= 37 539)**		1293 (3.4)	200 (0.5)	760 (2.0)	354 (0.9)
**Normal inflammatory markers^a^ (*n*= 98 951)**		5912 (6.0)	1652 (1.7)	2908 (2.9)	1503 (1.5)
**Raised inflammatory markers^b^ (*n*= 38 010)**		5712 (15.0)	2121 (5.6)	2407 (6.3)	1407 (3.7)
**Raised inflammatory markers, subdivided according to subsequent test results (*n*= 38 010)**	**No subsequent inflammatory markers done (*n*= 27 874)**	3471 (12.5)	1643 (5.9)	952 (3.4)	994 (3.6)
	**Subsequent inflammatory markers normal (*n*= 3314)**	615 (18.6)	249 (7.5)	321 (9.7)	70 (2.1)
	**One or more raised inflammatory marker on subsequent testing (*n*= 6822)**	1626 (23.8)	515 (7.6)	848 (12.4)	343 (5.0)

a All inflammatory marker tests normal.

b One or more inflammatory markers raised.

Of those with one or more raised inflammatory marker (*n* = 38 010), incidence of disease was highest among those with persistently raised inflammatory markers on subsequent testing, lower in those with normal inflammatory markers on subsequent testing, and lowest among those without subsequent testing in the next 90 days. Cancer was the notable exception; those with a normal subsequent test had a lower disease risk (2.1%) than those without repeat testing (3.6%).

**Figure 2.**
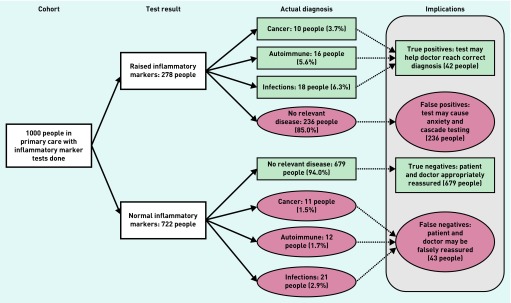
***Test implications flowchart. Numbers and percentages do not add up to 100% due to rounding and because a small number of people developed more than one relevant disease outcome.***

Figure 2 shows the incidence of disease according to test outcomes, simplified to a nominal population of 1000 tested patients. Of those with a positive test result, 85% had no evidence of infection, autoimmune condition, or cancer (false-positives).

Table 2 summarises the detailed performance characteristics of each of the three tests (CRP, ESR, PV) for the primary outcome of any relevant disease, as well as for each of the three disease outcomes separately. All three tests had overall sensitivities of <50% for the primary outcome, any relevant disease (defined as infections, autoimmune conditions or cancers).

**Table 2.** Performance characteristics of inflammatory markers for relevant disease, including infections, autoimmune conditions, and cancer

	**True-positive, *n***	**False-positive, *n***	**True-negative, *n***	**False-negative, *n***	**Sensitivity, % (95% CI)**	**Specificity, % (95% CI)**	**AUC, % (95% CI)**	**PPV, % (95% CI)**	**NPV, % (95% CI)**	**DOR^a^ (unadjusted) (95% CI)**	**DOR, (adjusted for age and sex) (95% CI)**
**CRP, *n*= 97 203**											
Any relevant disease	3947	18 745	69 797	4714	45.6 (44.5 to 46.6)	78.8 (78.6 to 79.1)	0.65 (0.64 to 0.66)	17.4 (16.9 to 17.9)	93.7 (93.5 to 93.9)	3.12^b^ (2.98 to 3.26)	2.86^b^ (2.73 to 2.99)

Infection	1780	20 912	72 279	2232	44.4 (42.8 to 45.9)	77.6 (77.3 to 77.8)	0.64 (0.63 to 0.65)	7.84 (7.5 to 8.2)	97.0 (96.9 to 97.1)	2.76^b^ (2.59 to 2.94)	2.74^b^ (2.57 to 2.92)

Autoimmune conditions	1404	21 288	73 063	1448	49.2 (47.3 to 51.1)	77.4 (77.2 to 77.7)	0.66 (0.65 to 0.67)	6.19 (5.88 to 6.51)	98.1 (97.9 to 98.2)	3.33^b^ (3.09 to 3.59)	3.05^b^ (2.83 to 3.29)

Cancer	915	21 777	73 360	1151	44.3 (42.1 to 46.5)	77.1 (76.8 to 77.4)	0.64 (0.63 to 0.65)	4.03 (3.78 to 4.30)	98.5 (98.4 to 98.5)	2.68^b^ (2.45 to 2.92)	2.04^b^ (1.86 to 2.23)

**ESR, *n*= 79 430**											
Any relevant disease	2780	15 589	57 221	3840	42.0 (40.8 to 43.2)	78.6 (78.3 to 78.9)	0.64 (0.64 to 0.65)	15.1 (14.6 to 15.7)	93.7 (93.5 to 93.9)	2.66^b^ (2.52 to 2.80)	2.43^b^ (2.30 to 2.56)

Infection	918	17 451	59 251	1810	33.7 (31.9 to 35.5)	77.3 (77.0 to 77.5)	0.58 (0.57 to 0.59)	5.0 (4.69 to 5.32)	97.0 (96.9 to 97.2)	1.72^b^ (1.59 to 1.87)	1.67^b^ (1.54 to 1.82)

Autoimmune conditions	1285	17 084	59 872	1189	51.9 (50.0 to 53.9)	77.8 (77.5 to 78.1)	0.70 (0.69 to 0.71)	7.0 (6.63 to 7.37)	98.0 (97.9 to 98.2)	3.79^b^ (3.49 to 4.10)	3.40^b^ (3.14 to 3.69)

Cancer	696	17 673	60 120	941	42.5 (40.1 to 45.0)	77.3 (77.0 to 77.6)	0.65 (0.63 to 0.66)	3.79 (3.52 to 4.08)	98.5 (98.4 to 98.6)	2.52^b^ (2.28 to 2.78)	2.07^b^ (1.87 to 2.30)

**Plasma viscosity, *n*= 13 834**											
Any relevant disease	536	3242	9439	617	46.5 (43.6 to 49.4)	74.4 (73.7 to 75.2)	0.63 (0.61 to 0.65)	13.9 (12.9 to 15.1)	93.9 (93.4 to 94.3)	2.53^b^ (2.23 to 2.86)	2.32^b^ (2.05 to 2.62)

Infection	183	3595	9767	289	38.8 (34.3 to 43.3)	73.1 (72.3 to 73.9)	0.58 (0.55 to 0.61)	4.84 (4.18 to 5.58)	97.1 (96.8 to 97.4)	1.72^b^ (1.42 to 2.08)	1.68^b^ (1.39 to 2.04)

Autoimmune conditions	234	3544	9834	222	51.3 (46.6 to 56.0)	73.5 (72.8 to 74.3)	0.65 (0.63 to 0.68)	6.19 (5.45 to 7.01)	97.8 (97.5 to 98.1)	2.92^b^ (2.42 to 3.53)	2.65^b^ (2.19 to 3.21)

Cancer	141	3637	9922	134	51.3 (45.2 to 57.3)	73.2 (72.4 to 73.9)	0.64 (0.61 to 0.68)	3.73 (3.15 to 4.39)	98.7 (98.4 to 98.9)	2.87^b^ (2.206 to 3.65)	2.38^b^ (1.86 to 3.03)

a Diagnostic odds ratio = the ratio of the odds of the test being positive if the subject has the disease relative to the odds of positivity in the non-diseased.

b P<0.001. AUC = area under the curve. CRP = C-reactive protein. DOR = diagnostic odds ratio. ESR = erythrocyte sedimentation rate. NPV = negative predictive value. PPV = positive predictive value.

### Inflammatory marker levels

**Figure 3.**
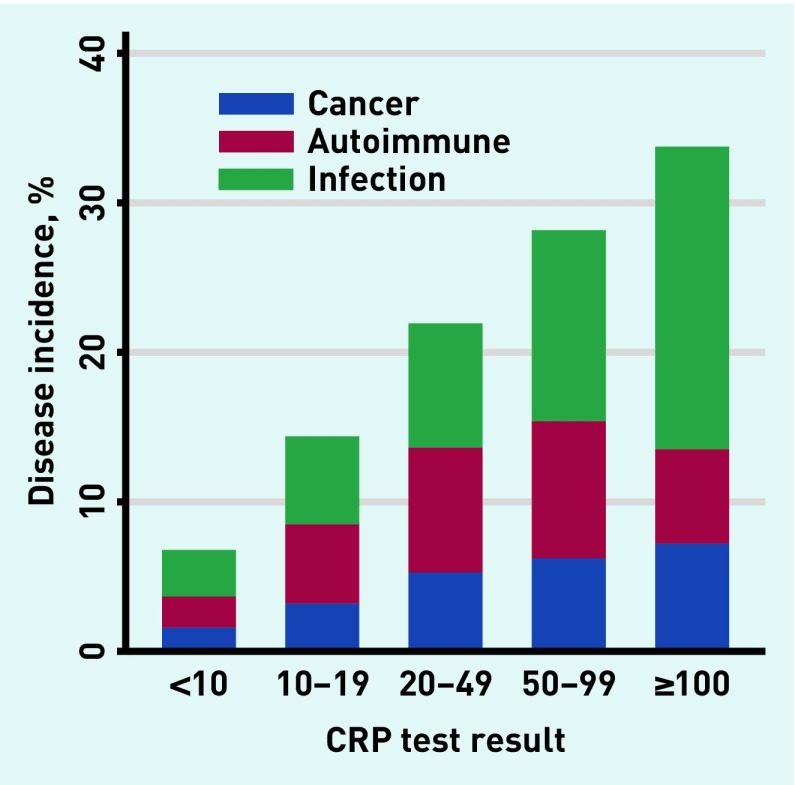
***Incidence of relevant disease in relation to test result for CRP. CRP = C-reactive protein.***

**Figure 4.**
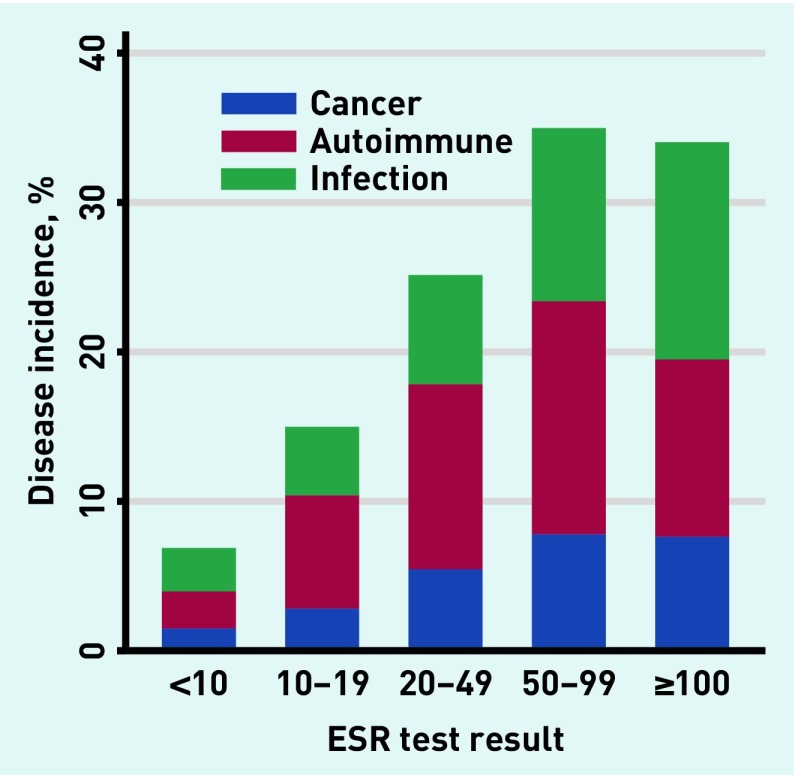
***Incidence of relevant disease in relation to test result for ESR. ESR = erythrocyte sedimentation rate.***

**Figure 5.**
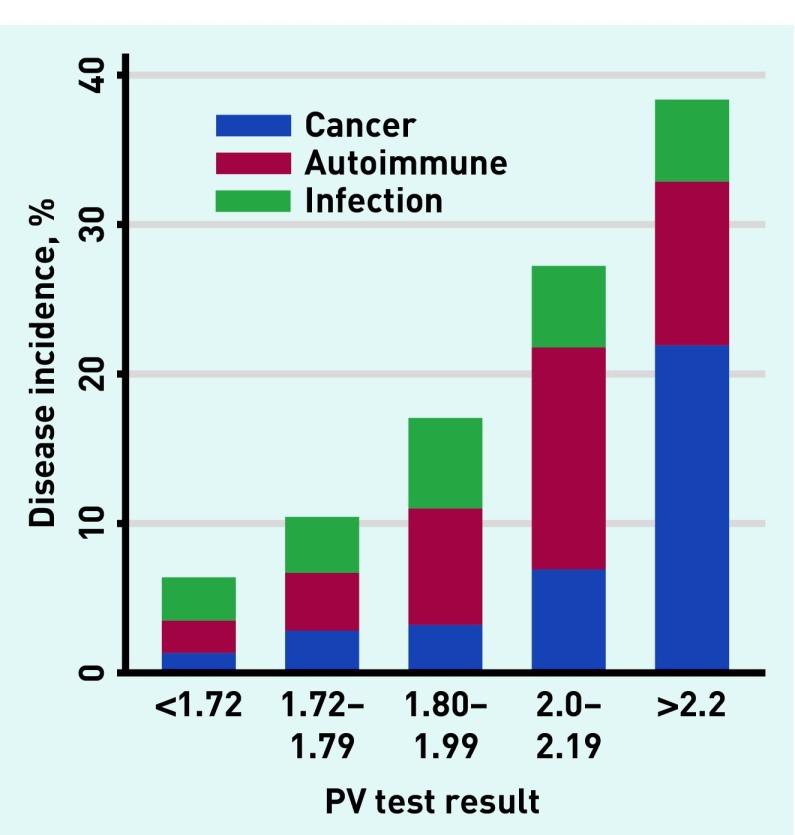
***Incidence of relevant disease in relation to test result for PV. PV = plasma viscosity. For the small number (<0.5%) with one or more disease outcome; cancer superseded autoimmune disease, which superseded infections.***

Incidence of disease increased with rising inflammatory marker levels in a dose–response relationship (Figures 3–5). With CRP ≥100 mg/L (*n* = 1552), 501 (32.3%) developed one or more relevant diseases: 113 (7.2%) cancers, 99 (6.4%) autoimmune conditions, and 317 (20.4%) infections. With ESR ≥100 mm/h (*n* = 389), 141 (36.3%) developed ≥one relevant diseases: 59 (15.2%) cancers, 60 (15.4%) autoimmune conditions, and 36 (9.3%) infections. With PV ≥2 Pa/s (*n* = 276), 81 (29.3%) developed one or more relevant disease: 30 (10.9%) developed cancer, 38 (13.8%) developed autoimmune conditions, and 15 (5.4%) developed infections.

### Symptoms triggering testing

Table 3 shows the most common symptoms in the 28 days before testing, ordered according to the ratio between symptom frequency in test-positive versus test-negative groups. Broadly these could be categorised into non-specific symptoms, abdominal symptoms, joint symptoms, and infective symptoms. Non-specific symptoms such as tiredness, dizziness, and low mood were relatively more common in the test-negative compared to test-positive groups, indicating that these non-specific symptoms are less likely to generate raised inflammatory markers. In comparison, infective symptoms such as cough, UTI, and chest infection were more likely to be associated with a raised inflammatory marker.

**Table 3.** Most frequently recorded symptoms in the 28 days before inflammatory marker testing

**Symptom**	**Untested (*N*= 37 539)**		**Normal inflammatory markers (*N =* 98 951)**		**Raised inflammatory markers (*N =* 38 010)**	
	***n***	**%**	***n***	**%**	***n***	**%**
Tiredness	82	0.22	6390	6.46	2148	5.65
Dizziness	126	0.34	2156	2.18	755	1.99
Headache	98	0.26	2498	2.52	965	2.54
Low mood	111	0.30	964	0.97	390	1.03
Low back pain	138	0.37	2079	2.10	857	2.25
Back pain	156	0.42	2202	2.23	964	2.54
Abdominal pain	166	0.44	7132	7.21	3232	8.50
Chest pain	100	0.27	1922	1.94	872	2.29
Rash	145	0.39	1663	1.68	797	2.10
Joint pain	52	0.14	2515	2.54	1215	3.20
Pain, generalised	87	0.23	1841	1.86	1011	2.66
Diarrhoea	64	0.17	2297	2.32	1266	3.33
Shoulder pain	123	0.33	1103	1.11	631	1.66
Throat symptoms	102	0.27	1018	1.03	598	1.57
Knee pain	182	0.48	1599	1.62	916	2.41
Nausea and vomiting	56	0.15	1171	1.18	720	1.89
Cough	496	1.32	3361	3.40	2336	6.15
Malaise	27	0.07	1005	1.02	720	1.89
UTI	189	0.50	1291	1.30	1057	2.78
Chest infection	130	0.35	720	0.73	804	2.12

Symptoms are ordered according to whether they were relatively more common in patients with normal inflammatory markers (top) or more common in those with raised inflammatory markers (bottom) UTI = urinary tract infection.

### Diagnostic activity after initial inflammatory marker test

Table 4 shows the blood tests, appointments, and referrals in the 6 months after testing for true-positive, false-positive, true-negative, or false-negative groups, plus untested controls. Follow-on blood tests, appointments, and referrals were higher in the false-positives than the true-negatives. Both groups consist of tested patients without subsequent pathology, with the main difference being the inflammatory marker result. Based on this, for 1000 inflammatory marker tests performed, the authors would expect 236 false-positives, associated with an additional 710 GP appointments, 229 phlebotomy appointments, and 24 referrals in the 6 months following testing.

**Table 4.** Cascade effects of testing in 6-month period after testing

	**Mean number of GP appointments per person^a^ (95% CI)**	**Mean number of phlebotomy appointments in 6 months (95% CI)**	**Mean number of total tests requested (95% CI)**	**Mean number of referrals per person (95% CI)**
True-positives, *n*= 5712	15.0^b^ (14.7 to 15.3)	4.26^b^ (4.15 to 4.37)	43.7^b^ (42.3 to 45.2)	0.86^b^ (0.83 to 0.89)
False-negatives, *n*= 5912	12.0^b^ (11.7 to 12.2)	3.23^b^ (3.14 to 3.32)	30.0^b^ (28.8 to 31.2)	0.78^b^ (0.75 to 0.81)
False-positives, *n*= 32 298	10.3^b^ (10.2 to 10.4)	2.78^b^ (2.75 to 2.82)	24.3^b^ (23.9 to 24.7)	0.62^b^ (0.61 to 0.64)
True-negatives, *n*= 93 039	7.29^b^ (7.2 to 7.3)	1.81^b^ (1.80 to 1.83)	13.7^b^ (13.5 to 13.8)	0.52^b^ (0.51 to 0.52)
Untested controls *n*= 37 539	4.80 (4.74 to 4.86)	1.14 (1.12 to 1.16)	9.66 (9.47 to 9.85)	0.24 (0.24 to 0.25)

True-positives = people with a positive test who develop relevant disease. False-negatives = people with a negative test who develop relevant disease. False-positives = people with a positive test with no relevant disease. True-negatives = people with a negative test with no relevant disease.

a Includes face-to-face consultations, home visits, and telephone consultations.

b P<0.001 — comparing true-positives to false-negatives and comparing false-positives to true-negatives.

## DISCUSSION

### Summary

The authors examined the outcomes of inflammatory marker testing in UK primary care. Multiple simultaneous inflammatory markers were common, and abnormal results frequent. Testing was more common in females, in white ethnic groups, and in the most affluent. Conversely, abnormal results were more common in patients from the most socially-deprived socioeconomic groups. This is in keeping with the inverse care law,^20^ with potential overtesting in the affluent, and relative undertesting in more deprived groups. Higher testing rates may also in part reflect higher consultation rates in certain sociodemographic groups.

Inflammatory markers have poor sensitivity, so are not suitable as a rule-out test. False-positive results were frequent, with increased rates of GP appointments, repeat blood tests, and referrals. In patients with a raised inflammatory marker, the most common diagnoses were infection (6.3%), followed by autoimmune conditions (5.6%), and cancers (3.7%).

### Strengths and limitations

The size of this study is a strength, along with the setting in primary care, where initial suspicion of disease usually arises. Examining multiple disease outcomes allowed the authors to explore the utility of testing to distinguish between healthy and unhealthy. This is relevant, as GPs describe using inflammatory markers in this way.^7^ The authors have also used a test consequences graphic, based on a nominal population of 1000 tested patients; this may help implementation into practice.^21^

The main limitation is lack of information about the reason for testing. The authors cannot determine which tests were done for specific diagnostic purposes, and which were done as a general rule-out for any relevant underlying disease. The frequency of non-specific symptoms in the cohort (tiredness, malaise, dizziness, or low mood) suggests the latter is likely to be common. The benefit of this approach is that it reflects real life clinical practice; though GPs may not have a specific diagnosis in mind when they request inflammatory markers, they need to consider a wide range of possible diagnoses if the test is positive.

All studies using electronic health records rely on the quality of data recording. Blood tests are electronically transmitted to the GP records, reducing the risk of missing or erroneous data. The authors used rigorous methods to develop disease code lists,^15^ but it is possible that there were some omissions. Furthermore, some diseases may be unrecorded, though this is rare for cancers and autoimmune disease.^22^ The authors omitted cardiovascular disease as an outcome; though CRP predicts future cardiovascular disease, it does not form part of any cardiovascular diagnostic algorithm. There is some risk of incorporation bias, particularly for infections, as these diagnoses may be more likely to be coded because of the inflammatory marker test result.

### Comparison with existing literature

Most previous studies of inflammatory markers consider single diseases, and most are based in secondary care. The current findings confirm the associations between inflammatory markers and infection and autoimmune conditions; however, the PPVs are lower in a primary care population with low prevalence for these conditions, and false-positives are more frequent.^3^

The cascade effects of medical technology have been described,^12^^,^^23^ yet there is limited evidence of the size of this effect.^14^ The authors identified a significant difference in the rate of GP consultations, blood tests, and referrals in the false-positive test result group.

Current guidelines for chronic fatigue, irritable bowel disease, and suspected dementia recommend inflammatory marker testing in order to exclude other diagnoses.^8^^–^10 The current research is discordant with these guidelines, showing that, with an overall sensitivity of <50% inflammatory markers are not a useful test of exclusion.

### Implications for research and practice

Previous qualitative work has shown that doctors perceive inflammatory markers as a useful rule-out test.^7^ However, the sensitivity of inflammatory markers is poor, so they are not suitable for that purpose. Instead they are classic Bayesian tests, with a positive test somewhat increasing the chance of disease, though not definitively, and a negative test reducing the chance of disease, but not to zero. The authors therefore suggest that inflammatory marker tests should not be used as a non-specific test to rule out disease, or for patient reassurance.

In patients with a raised inflammatory marker, the range of differential diagnoses is wide, explaining the additional consultations, tests, and referrals. With significantly raised inflammatory markers, the risk of disease is higher. In patients with unexplained raised inflammatory markers, risk of cancer must also be considered. Interpretation should take into account the reason for testing and the pre-test likelihood of disease; a negative test in the context of low-risk symptoms reduces disease likelihood further, but with the potential for harm from false-positive tests. False-negative tests may also lead to false reassurance, as patients with normal inflammatory markers are at significantly higher disease risk than untested controls. GPs should not be excessively reassured by a repeat negative inflammatory marker; the authors found higher disease incidence in those with normal repeat tests (18.6%) compared to those who did not have repeat tests performed (12.5%). Presumably this reflects the fact that the GP’s decision to repeat the test meant they were suspicious that the patient was ill.

Though the unit cost of inflammatory marker tests is relatively low, the total costs, including follow-on consultations, investigations, and referrals, are likely to be substantial. As well as financial costs, patient anxiety and GP workload may be generated. Further studies including health economic evaluations may be useful to inform clinical guidelines and recommendations for GPs about when (and when not) to use inflammatory marker tests. They should also consider whether specific inflammatory markers are superior in certain diagnostic scenarios.
